# The role of dysbiosis in shaping host immunity in endometrial cancer development

**DOI:** 10.3389/fimmu.2025.1627285

**Published:** 2025-09-19

**Authors:** Wiktoria Wierzbińska, Olga Kuźmycz, Aleksandra Kowalczyk, Paweł Stączek

**Affiliations:** ^1^ BioMedChem Doctoral School of the University of Lodz and Lodz Institutes of the Polish Academy of Sciences, Lodz, Poland; ^2^ Department of Molecular Microbiology, Institute of Microbiology, Biotechnology and Immunology, Faculty of Biology and Environmental Protection, University of Lodz, Lodz, Poland

**Keywords:** endometrial cancer, endometrial microbiota, dysbiosis, tumor microenvironment, estrabolome, gut microbiota

## Abstract

In recent years, research into the background of carcinogenic processes has increasingly focused on the role of the tumor microenvironment (TME) in tumorigenesis. In addition to the presence of tumor cells and non-malignant components, which include immune cells, extracellular matrix elements, stroma, and endothelial cells, the microbiome is now increasingly being classified as an integral part of the TME. The establishment of the Human Microbiome Project (HMP) in 2007 along with the development of next-generation sequencing (NGS) techniques proved to be a breakthrough in terms of human microbiota research, shedding new light on the existing knowledge of microorganisms inhabiting various niches of the human body and their functions. Emerging scientific evidence from preclinical and clinical studies indicates significant differences in the microbiome composition between tumor tissues and benign controls. The presence of specific pathogenic strains within a tissue may play a key role in the initiation and progression of inflammation, which not only may be directly responsible for the stimulation of tumorigenic processes but may also affect the destabilization of the host genome, causing significant disruption of its metabolism. The role of microorganisms in the induction and promotion of pathological processes, including cancer, has been confirmed in many studies to date. Recent years of research on the microbiota of the female reproductive tract (FRT) have not only indicated that the endometrium has its unique microbial composition but have also made it possible to point out differences in composition between the microbiome of healthy and tumor-lesioned tissue, suggesting a potential role for dysbiotic disorders in the pathogenesis of endometrial cancer (EC). In this review, we aim to highlight the complex interplay between bacterial interactions and host immunity, and how this phenomenon contributes to the development and progression of endometrial cancer.

## 1 Introduction

For almost a century, due to the work of Henry Tissier from 1900, the female upper reproductive tract (URT) was considered sterile (1). The presence of bacteria in this area has been linked to pathologies and the development of disease states. In terms of sterility, the FRT was divided into three sectors, the non-sterile vagina, the cervical mucus plug, which provides a barrier to microorganisms, and the sterile endometrium and fallopian tubes (2). That was until the second half of the 20th century, when the “sterile endometrium” theory was increasingly challenged by case reports of patients in whom the presence of microorganisms in the uterine cavity was confirmed using culture methods (3–15). Unfortunately, the majority of these studies were performed on the material obtained from swabs, which posed a significant risk of contamination. Moreover, studies based on culture methods did not reflect the biodiversity of microorganisms inhabiting the uterine cavity, as only 1% of bacteria residing in the human body can grow on synthetic media (16).

The development of modern sequencing techniques, including those based on metagenomic analyses, allowed the identification of tested material to be carried out with greater efficiency, less expense and higher precision than in the case of cultivation methods (17). It is becoming clear that each part of the URT harbors its unique microbiome. Microbial balance within the female URT is a key element of the symbiotic relationship between microorganisms and the host. Microbial homeostasis is guarded by the immune system, which maintains a certain limit of invasion of commensal microorganisms and eliminates pathogens. Interactions between immune cells, cytokines and hormones are important factors enabling the maintenance of mucosal homeostasis. However, when the microbial load is too high or rich in representatives of certain taxa, excessive tissue destruction and immune stimulation may occur, leading to an imbalance of homeostatic relationships between bacteria. It is not known whether dysbiosis is a cause or effect of the disease, but disorders within the composition of the endometrial microbiome are associated with the occurrence of abnormalities in the physiology of the FRT (18). Dysbiotic alterations within the female URT microbiome represent a bacterial factor that is increasingly considered in the context of a potential risk factor for cancerous lesions. Disruption of microbial homeostasis can cause chronic inflammation, leading to a variety of immunopathologies and tumor-inducing diseases. Microbes can also promote the destabilization of the host genome by releasing toxins that can damage its genetic material. The presence of certain bacterial species can contribute to altering the expression of genes encoding proteins involved in the inflammatory response, proliferation, apoptosis, and modification of the secretion of inflammatory factors. Consequently, these events can ultimately disrupt physiological processes and promote the development of disease states, including cancer (19, 20).

## 2 Endometrial microbiota representatives and origin

### 2.1 The origin of endometrial microbiota

**Figure 1:**
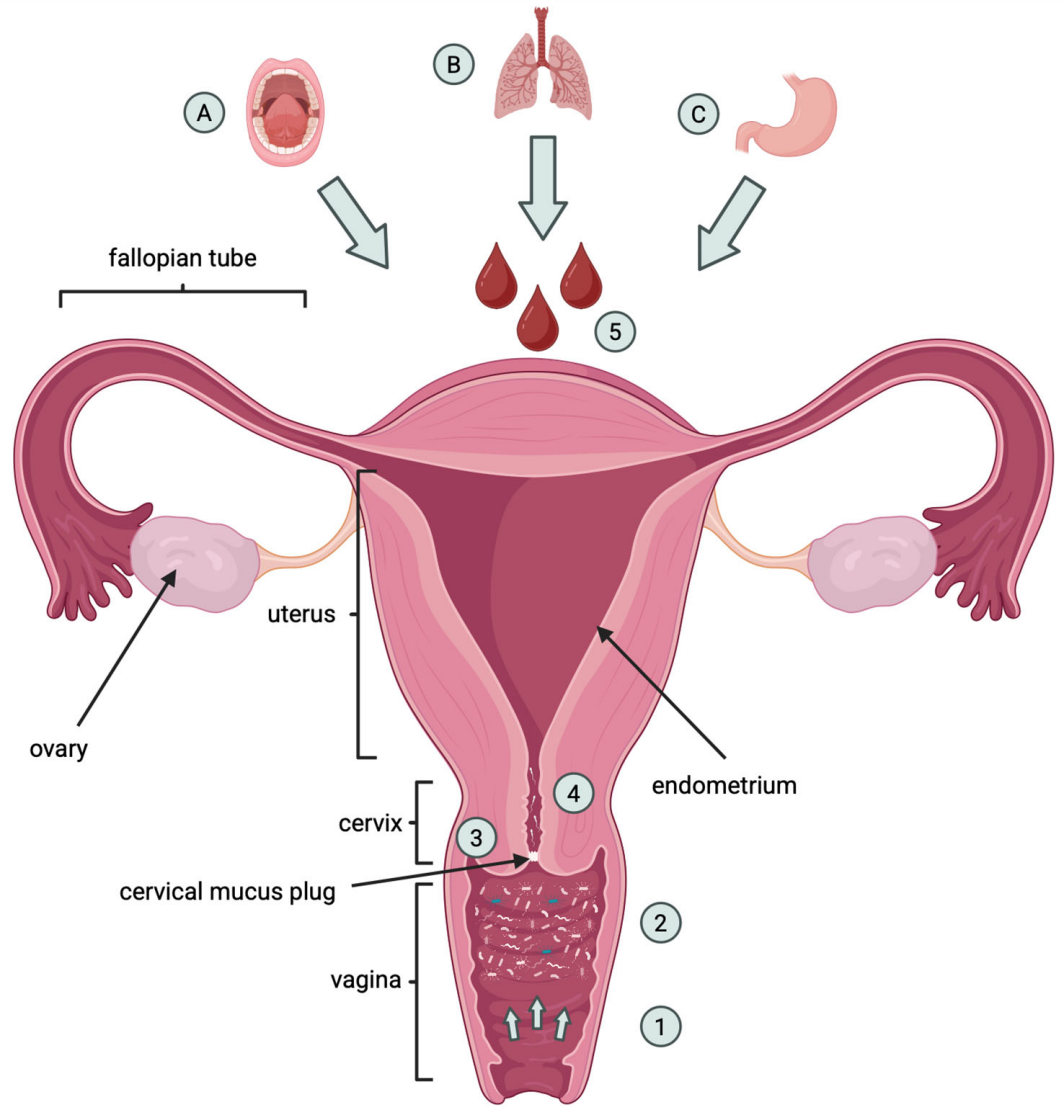
The origin of endometrial microbiota. The diagram illustrates the potential sources of microorganisms colonizing the endometrial environment, highlighting the role of the anatomical, mechanical and haematogenic factors. 1 – Dysfunction of uterine peristaltic activity can promote colonization through enhanced transport caused by increased frequency of contractions. 2 – Microorganisms entering from the vagina. 3 – The cervical mucus suppository limits but does not completely block the passage of microorganisms from the vagina 4 – The peristaltic pump supports the transport of sperm cells from the cervix, which may be accompanied by a movement of other factors, including bacteria. 5 – Endometrium can also be colonized by microorganisms spread by blood that originate from the oral cavity **(A)**, respiratory tract **(B)** or gastrointestinal tract **(C)**. Created in BioRender. Wierzbińska, W (2025). https://BioRender.com/xmrvrud.

The most likely source of microorganisms colonizing the endometrium is bacteria ascending from the vagina (**Figure 1**). This hypothesis is supported by the study of Swidsinski and colleagues (21), who showed the presence of biofilms of *Gardnerella vaginalis* on the endometrium and fallopian tubes in patients suffering from bacterial vaginitis. Subsequent years of analysis have shown that the cervical mucus plug, previously considered an impermeable barrier separating the vagina from the endometrium for bacteria, is not effective against all microorganisms. A study by Hansen and colleagues (22) found *Ureaplasma parvum* bacteria to be present within it. The researchers suggested that this barrier significantly restricts the passage of microorganisms from the vagina to the lower pole of the uterus, but does not block it completely, so the endometrial sterility theory has once again been challenged. Colonization of the endometrium can also be supported by physiological uterine contractions. The peristaltic pump promotes the translocation of sperm from the cervical canal, which may be accompanied by the movement of other particles, including bacteria, especially during the follicular and luteal phases of the cycle, when contractions intensify (23, 24). Dysfunction of uterine peristaltic activity may particularly favor colonization through increased transport caused by increased frequency of contractions (25). Some studies suggest that in addition to bacteria representing the vaginal microflora, the endometrium may also be colonized by blood-spreading microorganisms from the respiratory tract, gastrointestinal tract, or oral cavity (26). It has been shown that bacteria commonly colonizing the upper respiratory tract, such as *Haemophilus influenzae* or *Streptococcus pneumoniae*, can also colonize the female reproductive system, with the consequence that pregnancy can be terminated prematurely (27). Results of another study showed a similarity between the placental microbiome and oral microbiome of nonpregnant women (28). Among obese women with common disorders of the intestinal microbiome, a disproportion was more often observed among the population of microorganisms colonizing the vagina, which could result in preterm labor (29).

### 2.2 Representatives of endometrial microbiota

Similar to the vaginal microbiome, it is commonly believed that the endometrium of healthy women is a niche dominated by bacteria of the genus *Lactobacillus*. This thesis is supported by numerous studies that have shown the predominance of lactobacilli over other microorganisms residing in the uterine cavity. Results obtained by the team of Mitchell et al. (3) revealed that the species most frequently isolated from sterile endometrial swabs in a group of 58 women undergoing hysterectomy were *Lactobacillus iners*, *Prevotella* spp. and *Lactobacillus crispatus*. The study, led by Franasiak (4), found a predominance of microorganisms from the genera *Flavobacterium* and *Lactobacillus* in the material collected from 33 patients. Miles (5) also identified lactic acid bacilli as the microorganisms most frequently isolated from the endometrium of patients undergoing hysterectomy. Moreno et al. (6), based on a study of endometrial fluid collected from 52 women revealed that the endometrial cavity was most abundantly colonized by bacteria of the genus *Lactobacillus*, followed by *Gardnerella*, *Bifidobacterium*, *Streptococcus* and *Prevotella*. Based on the results of their own experience, supported by available literature sources, the researchers proposed a method for classifying the microorganisms residing in the uterine cavity, distinguishing a microflora dominated by *Lactobacillus* (LD), which is one in which bacteria of this genus make up more than 90% of the population, and NLD, where the dominance of lactic acid bacilli over other species is less. Tao et al. (8, 16) observed predominance of lactic acid bacilli oscillating between 70-90% relative to other taxa. The dominance of bacteria of the genus *Lactobacillus* within the endometrial microbiota was also confirmed by Wee and colleagues (8), who showed that the uterine cavity is characterized by a relatively lower microbial abundance compared to the vaginal microflora. Research on the endometrial microflora was also undertaken by Bednarska-Czerwińska (9), who identified 22 microbial species within it with a definite dominance of the genus *Lactobacillus* over other taxa, as well as a team of Lüll and colleagues (10), who also demonstrated a predominance of lactobacilli in endometrial fluid and tissue fragments collected from 25 patients.

Despite the prominent results, it is still hard to determine the definite molecular pattern of microorganisms residing in healthy endometrium. Some studies have found no lactobacilli in the reproductive tract of healthy, asymptomatic women, which may suggest that the dominance of lactobacilli over other microorganisms residing in the endometrium is not always associated with a condition indicative of abnormalities. Verstraelen and colleagues (11), in a study conducted on a group of 19 healthy women, showed a dominance of *Bifidobacteria*, while lactic acid bacilli prevailed in only four cases. *Pseudomonas* spp., *Acinetobacter* spp., *Vagococcus* spp. and *Sphingobium* spp. were the dominant species among the microorganisms isolated from the endometrium by the team of Chen and colleagues (12). Similar results were obtained by Winters (13), who identified *Acinetobacter, Pseudomonas, Cloacibacterium, and Comamonadaceae*. In shotgun sequencing conducted by the team of Li and colleagues (14), *Pseudomonas* sp., *Propionilbacterium* sp., *Streptococcus* sp., and *Moraxella* sp. were described as dominant taxa. The study by Sola-Leyva (15) showed a predominance of *Klebsiella pneumoniae*, *Clostridium botulinum* and *Pasterurella multocida*, over other species isolated from the endometrium.

The reasons for the differences in the results obtained can be traced to physiological stimuli at the individual level, as well as technical ones, resulting from the methodology of the study. There are many factors, such as medical history, endocrine disorders, genetic conditions, age, ancestry or behavioral variables, that can directly and indirectly affect the composition of a patient’s microbiota. Studies have shown that the microbiome of women suffering from various types of reproductive disorders, such as endometriosis, endometrial cancer, polyps, or infertility, had a different composition than that observed in patients without similar abnormalities (30). It has also been shown that the abundance and biodiversity of microorganisms inhabiting the female reproductive system are subject to changes throughout a woman’s life and are significantly reduced during the menopausal period (31). Some studies also suggest the role of ethnicity as a potential factor affecting the composition of the microbiota of the female URT. Fettweis et al. (32) showed that the microbiota of women of European descent was significantly less species-diverse and poorer than that of African-American women. Zhou et al. (33) reported that the types of vaginal communities found in Japanese women were similar to those found in both African-American and European women. However, with few studies and significant differences in results, it is difficult to say whether ethnicity, rather than geographical area and the resulting behavioral and cultural differences may have a significant impact on the composition of the female reproductive tract microbiome. Changes in the composition of the female reproductive tract microbiome may also be caused by certain physiological conditions, such as pregnancy (34). Methodological and technical factors can also contribute to the discrepancy in results. One of the biggest obstacles in attempting to determine the composition of the endometrial microbiota is the limited ability to obtain high-quality material for testing. Most experiments are conducted on swabs taken from patients through the cervical canal, which carries a significant risk of contaminating samples with vaginal bacteria (35). Although studies conducted on tissues from patients undergoing hysterectomy have a lower risk of contamination, the composition of the microbiota of such patients, may differ from the physiological one, as such women have abnormalities, pathological conditions and diseases that may affect it (3). Another major limitation of most studies is the difficulty in obtaining study material, resulting in small sample sizes and reduced statistical value of the results (36). While the development of 16S rRNA variable regions (V) sequencing techniques has allowed for much more accurate studies of the human microbiome, this method also has its limitations. Available research protocols vary in terms of the amplification primers, sequencing primers or sequencing techniques used, which can contribute to discrepancies in the results obtained (37). In addition, the detection of bacterial genetic material is not always indicative of the presence of a viable microorganism in a given niche (38). An additional factor potentially explaining inconsistencies in reported prevalence of *Lactobacillus* dominance in the endometrial microbiota is the concept of functional redundancy among bacterial taxa. Even when *Lactobacillus* sp. is not taxonomically dominant, taxa such as *Gardnerella* and *Bifidobacterium* may perform analogous functions such as lactic acid production, modulation of pH, or competitive exclusion of pathogens, thereby preserving endometrial homeostasis. Inclusion of this functional perspective could help reconcile discordant findings across studies (16). Due to inconsistencies in the results obtained and the number of variables that must be considered when interpreting them, we cannot yet unequivocally define the composition of a healthy endometrial microbiota. However, based on the available data, we can make some generalizations. The microorganisms statistically most common in the endometrial cavity are bacteria belonging to types: *Bacteroidetes*, *Proteobacteria*, *Actinobacteria* and *Firmicutes*, among which the genus *Lactobacillus*, whose dominance within the endometrium has been confirmed by numerous research groups, deserves special attention. Lactobacilli are also a major component of the bacteria found in the vagina, but although the composition of the two habitats has some similarities, they are not identical. The endometrium has its unique microbiota (39).

## 3 Maintaining endometrial eubiosis

### 3.1 Eubiosis – the microbial homeostasis

It may seem impossible for the microorganisms to survive in this highly variable environment due to the menstrual cycle, but research clearly indicates that the endometrium has its own microbiota. Maintaining a balance between commensal bacteria and pathogens in this particular niche is essential for its healthy activity. Based on the studies of gut microbiota, in 2010, Hooper and Macpherson (40) described three types of immune barriers needed to maintain a balance between the gut microbiota and the host. The first barrier is the anatomical limitation of the exposure of commensal microorganisms to the host immune system. The next barrier is the immune mediators that limit direct contact of microorganisms with the epithelium, and the last one is the ability to rapidly detect and inactivate pathogenic microorganisms once any of the barriers are broken. Each of the above-mentioned conditions is initially fulfilled by the endometrial environment. The microbial homeostasis of the endometrium is guarded by the immune system, which takes care to maintain a certain limit of invasion of commensal microorganisms and elimination of pathogens (41).

One potential role of the endometrial microbiota is to modulate immune responses within the niche through several mechanisms, such as competition, receptor stimulation and modulation of epithelial barrier function. Both endometrial stroma cells and epithelial cells are equipped with several receptors that allow them to recognize foreign molecular patterns of pathogenic microorganisms. Recognition of foreign antigens leads to the activation of the inflammatory response, resulting in the secretion of a wide range of specific chemokines that are sensed by appropriate subsets of immune cells. They also stimulate the synthesis of PRR receptors. Moreover, commensal microorganisms restrict the pathogen invasion by depleting the environment of nutrients (42). The uterine cavity microbiome may also contribute to supporting the genomic stability of the epithelium by modulating transcription factors (30). Microbes can also strengthen the physical barrier of the endometrium by stabilizing the mucosal layer, promoting repair processes or secreting antimicrobial substances (43). The endometrial microbiome can also influence the phenotype of immune cells in the uterine cavity, which, in addition to their defensive role, play a key role in embryo implantation by providing local immune tolerance to both fetal and paternal antigens, as well as trophoblast invasion (44).

### 3.2 URT as an immunologically suited niche

**Figure 2:**
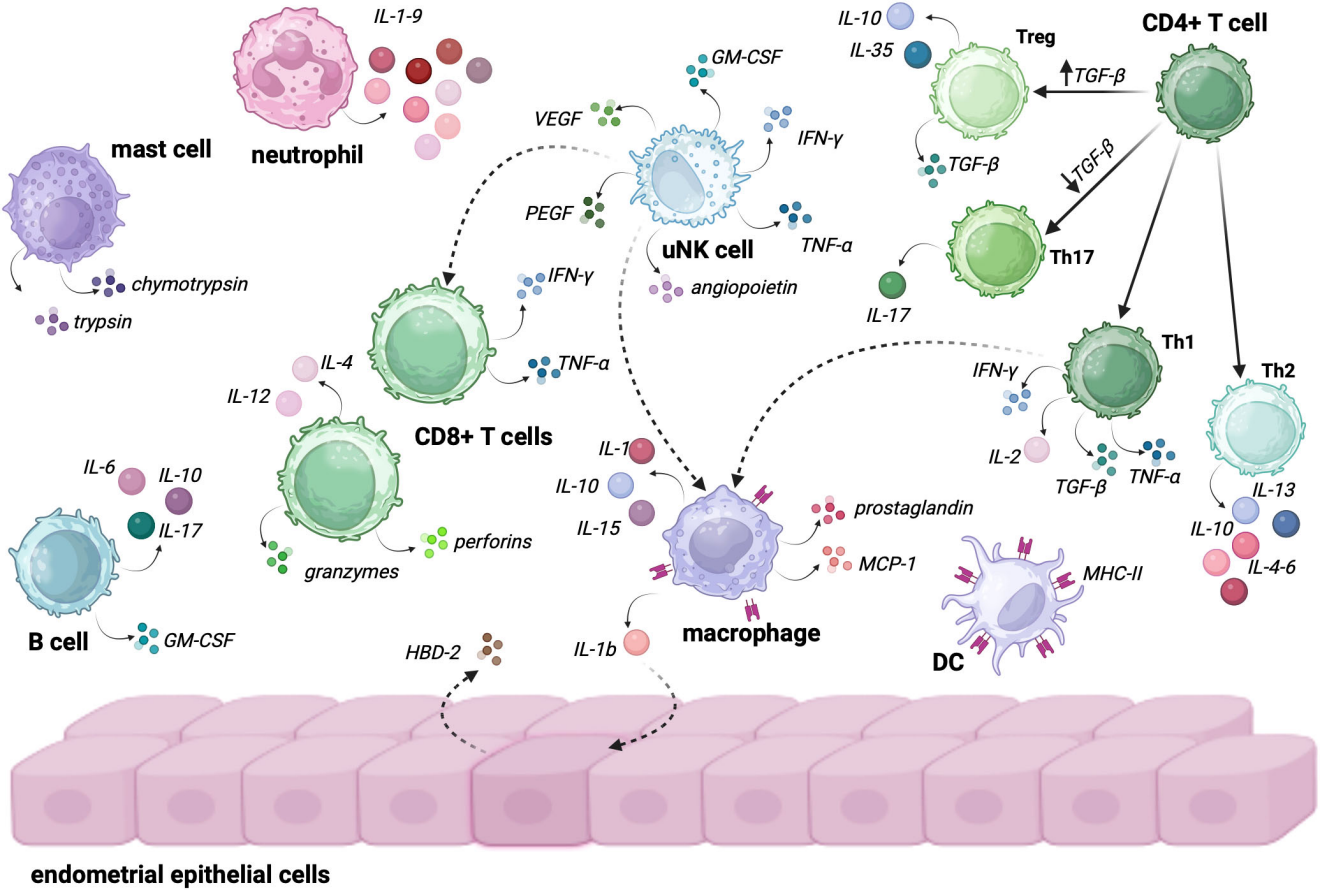
Endometrial immune cells and their role in sustaining immunological homeostasis. The interaction between these cells plays a crucial role in maintaining a balanced immune environment, which is essential for both fertility and successful pregnancy. • Neutrophils secrete IL-1-9, which contributes to early inflammation and defense against pathogens while also influencing the recruitment of other immune cells to the site of infection or tissue injury. • Mast Cells: secrete trypsin and chymotrypsin, enzymes that participate in tissue remodeling and inflammatory responses. Their role is particularly important in regulating vascular permeability and mediating allergic or immune responses within the endometrium. • B cells within the endometrial immune microenvironment secrete IL-6, IL-10, IL-17, and GM-CSF. These cytokines play diverse roles, including modulating local immune responses, regulating inflammation, and supporting the survival and function of other immune cells, such as macrophages and T cells. • uNK cells are central to the endometrial immune response, secreting a variety of factors, including VEGF, PEGF, angiopoietin, TNF-α, IFN-γ, and GM-CSF. These cytokines and growth factors are crucial for angiogenesis, tissue remodeling, and activating other immune cells like macrophages and CD8+ T cells. • CD8+ T Cells secrete IFN-γ, TNF-α, perforins, granzymes, IL-4 and IL-12, participating in immune surveillance and destroying infected or altered cells. Their activation is modulated by uNK cells and other immune cells, contributing to the balance between defense and immune tolerance. • Macrophages of the endometrium secrete IL-1, IL-10, IL-15, prostaglandins, MCP-1 and IL-1β, which influence local inflammation, tissue repair, and the recruitment of other immune cells. Macrophages also act as key regulators of the immune response, being able to activate endothelial cells to produce HBD-2, an antimicrobial peptide that further contributes to local immune defense. • DCs expressing MHC-II play a crucial role in antigen presentation and the initiation of adaptive immune responses. Their interaction with T cells helps to shape the endometrial immune landscape, promoting tolerance or active immune defense depending on the tissue requirements. • CD4+ T cells grouped into four subtypes are pivotal in regulating immune responses: o Treg cells are induced when TGF-β levels in the environment are high; they secrete IL-10, IL-35 and TGF-β, ensuring immune tolerance and preventing excessive inflammation. ○ TH17 are being activated when TGF-β levels are low; TH17 cells secrete IL-17, playing a role in pro-inflammatory responses and the recruitment of neutrophils and other immune cells. ○ Th1 secrete IFN-γ, IL-2, TGF-α, and TGF-β. These cells are involved in promoting immune defense and activating macrophages to clear pathogens or damaged cells. ○ Th2 secrete IL-13, IL-10, and IL-4, promoting tissue repair and helping to modulate the immune response to avoid excessive inflammation, contributing to the maintenance of homeostasis. Created in BioRender. Wierzbińska, W. (2025) https://BioRender.com/4xl6gb7.

The immune system plays a crucial role in maintaining homeostasis within the female URT, protecting it from pathogens and preventing pathological changes (**Figure 2**). Although immune cells are distributed differently across various regions of the reproductive system (**Table 1**), the most abundant are T lymphocytes, dendritic cells (DCs), natural killer (NK) cells, neutrophils, and mast cells which collectively orchestrate tissue remodeling, immune surveillance, and regulation of local inflammation, particularly during the secretory phase of the menstrual cycle. Dominance of these particular immune cells within the endometrium suggests that the immune landscape of this niche is predominantly shaped by cellular immunity, with innate immune cells constituting the majority of leukocyte populations. In contrast, humoral immunity plays a supplementary role, with low numbers of B lymphocytes and plasma cells producing limited amounts of secretory IgA and IgG in the endometrial milieu. This cellular predominance ensures rapid and localized immune responses while maintaining immune tolerance required for successful implantation (45).

**Table 1:** Immune cells crucial to function of endometrium.

Immune cell type	Abundance	Functions
uNK cells	most abundant in endometrium (70% in early pregnancy)	low cytotoxicity, cytokines secretion (IFN-γ, TNF-α), production of proangiogenic factors (VEGF, PlGF)
Macrophages	~10% of immune cells in endometrium	phagocytosis, tissue remodeling, cytokine secretion (IL-10, MCP-1), promote implantation
Dendritic Cells (DCs)	~10% of immune cells in endometrium	antigen presentation, phagocytosis, activation of T-cells, regulation of mucosal immunity
T Lymphocytes	third most abundant cells in endometrium	CD8+ (cytotoxic): induction of apoptosis, cytokines secretion; CD4+ (helper): coordination of immune response
Neutrophils	low abundance in endometrium, rises during menstruation	tissue remodeling during menstrual cycle, cytokines secretion: IL-1-9, phagocytosis, antimicrobial peptides synthesis
Mast Cells	low abundance in endometrium, near endometrial blood vessels	proteases release (trypsin, chymotrypsin) for inflammatory responses, biological defense
B Lymphocytes	rare (1% of endometrial immune cells)	cytokine secretion (IL-6, IL-10, IL-17), differentiationinto plasma cells

#### 3.2.1 Uterine natural killer cells

The most abundant immune cells residing in the endometrium are the uterine natural killer cells (uNK cells). The number of cells in the endometrium undergoes changes related to the menstrual cycle, increasing during the secretory phase. Also, the quantity of uNK cells rises in early pregnancy, representing up to 70% of all hematopoietic cells and gradually decreases in the middle and late gestation (46, 47). uNK cells are distinguished by lower cytotoxicity, fewer receptors responsible for activation and an increased number of inhibitory receptors compared to peripheral blood NK (pNK) cells, which may be related to the role of these cells in successful implantation (48). Moreover, the array of cytokines secreted by uNK cells differs from the mediators secreted by pNK. In addition to interferon γ (IFN-γ), tumor necrosis factor α (TNF-α), or granulocyte-macrophage colony-stimulating factor (GM-CSF), uNK are also capable of secreting proangiogenic factors like vascular endothelial growth factor (VEGF), placental growth factor (PlGF), and angiopoietin 2 (49, 50). In addition, uNK cells can modulate not only local inflammatory responses by producing inflammatory mediators but also induce and activate macrophages and cytotoxic T cells (51). Unfortunately, there is a lack of studies that clearly indicate what effect menopause has on the number and phenotype of uNK cells, however, the number of pNK cells decreases with age, which may suggest a similar relationship for cells in the uterine cavity (52). Given the diverse results of previous studies, the effect of age on NK cell cytotoxicity also remains unclear (53–55).

#### 3.2.2 Macrophages and dendritic cells

Macrophages and dendritic cells (DCs) represent the major antigen-presenting cells (APCs) in the uterine environment, accounting for about 10% of all endometrial immune cells. APCs are one of the key elements activating the inflammatory response by the bacterial molecular pattern and presentation of their antigens to T lymphocytes (56). Both macrophages and uDCs can be activated by detecting pathogens in the endometrium and starting the process of phagocytosis, internalization, and degradation of the antigen components. They present bacterial peptides to T-cells via major histocompatibility complex (MHC) receptors, which activate the T-cells to initiate a cell-mediated and/or humoral immune response via MHC class II molecules (57). DCs are the most effective antigen-presenting cells involved in the adaptive immune response activation and mucosal surface immune response regulation. Like macrophages, they are capable of phagocytosis, and thanks to lysosomal enzymes in phagosomes and lysozymes, they can destroy ingested pathogens (58).

Macrophages are the most abundant group of endometrial APCs. Their physiological role in the endometrium is associated with tissue clearance and remodeling (59). Moreover, together with uNK cells, macrophages are the major cytokine producers in the endometrium. They secrete IL-10, IL-15, IL-1, prostaglandin (PG), and monocyte chemoattractant protein-1 (MCP-1), which induce immunosuppression, differentiation and recruitment of other immune cells (60). Macrophages also have outstanding phagocytic abilities, enabling them to recognize foreign antigens. Bacterial lipopolysaccharide (LPS) stimulates IL-1b secretion by macrophages, which results in the secretion of human beta-defensin-2 (HBD-2) by cells of the endometrial epithelium to inhibit pathogen invasion (61). Macrophages also play an important role in the early stages of pregnancy. After fertilization, their number increases rapidly and decreases around the second trimester. In addition to providing the fetus with protection from infection, macrophages are involved in spiral artery remodeling, trophoblast invasion, and implantation (50).

#### 3.2.3 Neutrophils

Neutrophils’ presence in the endometrium increases innate immune defense thanks to the secretory capacity of IL 1-9, phagocytosis, production of oxidative compounds and release of antimicrobial peptides (62).

#### 3.2.4 Mast cells

The main role of endometrial mast cells is related to biological defense mechanisms by releasing proteases such as trypsin and chymotrypsin to activate inflammation and the immune response (63).

#### 3.2.5 B and T lymphocytes

In a healthy pre-menopausal endometrium, B cells are relatively rare, accounting for only 1% of immune cells. They participate in immune regulation by secreting a wide range of cytokines, including IL-6, IL-10, IL-17, and GM-CSF, and they can differentiate into plasma cells when stimulated by antigens (64). The main representatives of the adaptive immune response and the third most abundant endometrial immune cells are T cells. Uterine T cells consist of CD8+ (cytotoxic) cells, which make up 66% of this population, and CD4+ (helper) cells, which make up 33% of it. CD4+ T cells population is divided into Th1 cells (5% - 30%), Th2 cells (5%), Tregs (5%) and Th17 cells (2%) (65).

The main role of cytotoxic T cells is to participate in the apoptosis of the target cell. CD8+ T cells can directly initiate apoptosis by secreting cytotoxic perforin proteins and granzymes or by integrating the FasL-CD95 ligand (FasR), resulting in the initiation of apoptosis through the activation of caspase proteases in the intruder cell. In addition to participating in indirect apoptosis, CD8+ T cells can kill target cells by releasing cytokines such as TNF-α, IFN-γ, IL-4, and IL-12, which activate other immune cells in addition to affecting the target cell (66). During menstruation, endometrial CD8+ T cells can maintain cytolytic activity during the proliferative phase, but activity wanes during the secretory phase (67). In contrast, during early pregnancy, to maintain immune tolerance to fetal antigens and prevent infection, cells under the influence of hormones reduce the expression level of cytotoxic molecules (68).

CD4+ T cells represent a less abundant, however important, group of cells involved in the production of immune mediators and interaction with other immune cells upon activation by APCs. Th1 cells are responsible for the secretion of IFN‐γ, TNF‐β, IL‐2 and macrophage activation. Moreover, they also produce TNF-a, which is involved in promoting inflammation. Th2 cells secrete IL-4, IL-5, IL-6, IL-10 and IL-13, which are mostly involved in humoral immunity and extracellular pathogen infection (69). Th1 and Th2 cells mutually inhibit each other. Th2s are responsible for secretion of IL-10, a cytokine which, by influencing APCs, inhibits the development of Th1. In turn, Th1 cells produce IFN‐γ, which prevents Th2 cells from being activated (70). T17 cells are responsible for the secretion of the pro-inflammatory cytokine IL-17. Like Treg cells, they arise from naive CD4+ cells under the influence of TGF-β. Depending on the concentration of cytokine in the microenvironment, naive CD4+ cells differentiate into T17 cells when cytokine concentration is low or Treg cells when cytokine concentration is high (71). The main role of Treg cells is to reduce inflammation, crucial for the phenomenon of immune tolerance. Immunosuppression occurs when the TCR complex on the surface of Treg cells recognizes the peptide MHC class II (MHCII) on the surface of APC cells, resulting in Treg activation and an increase in their suppressive function. Treg cells express their function by secreting a wide range of immunosuppressive cytokines, including IL-10, IL-35 and TGF-β. In addition, Treg cells have the ability to uptake pro-inflammatory IL-2 from the environment due to high expression of the IL-2 surface receptor (72).

### 3.3 Menstrual cycle – hormonal dynamics and reproductive function

**Figure 3:**
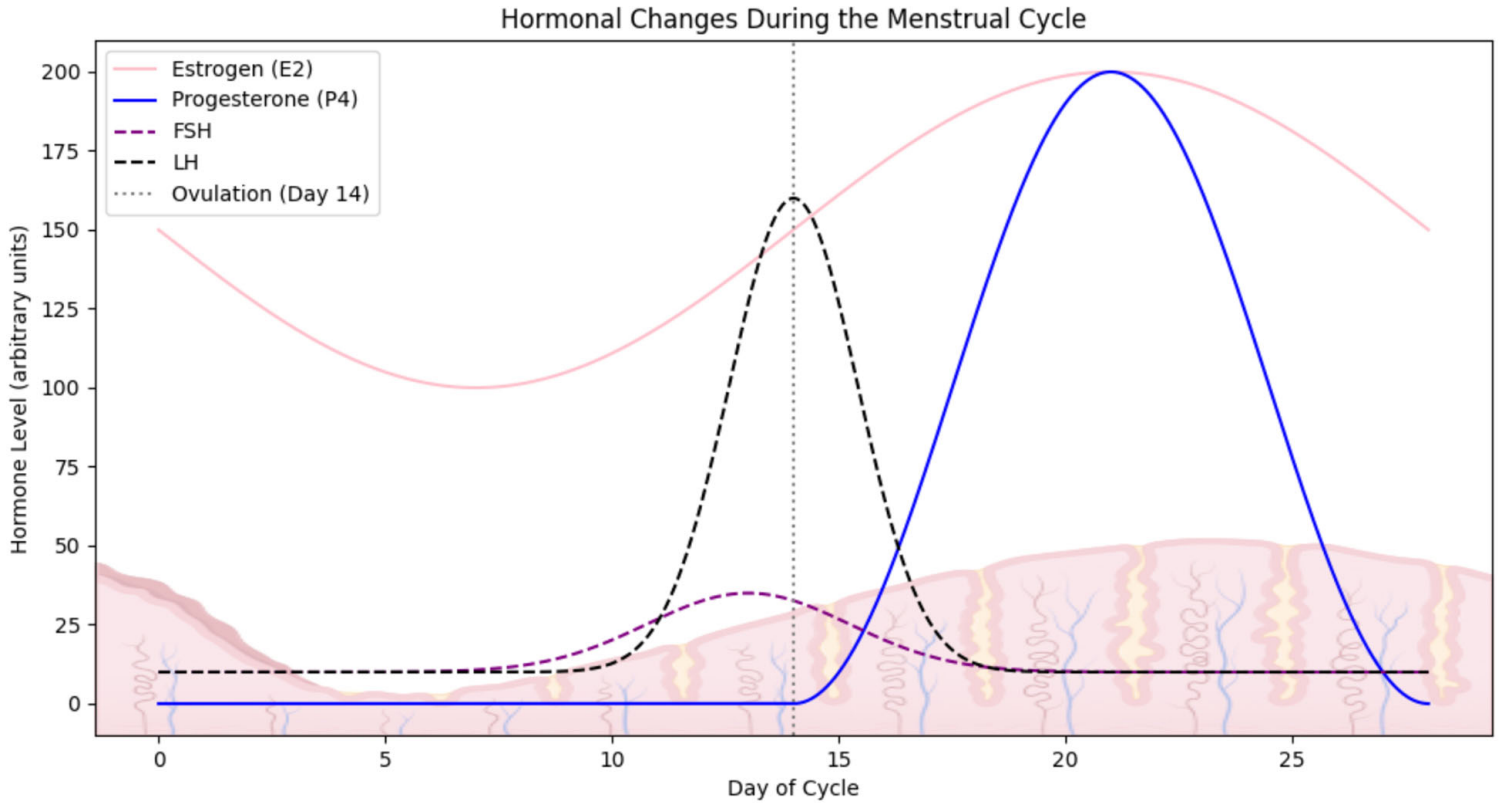
Hormonal fluctuations during the menstrual cycle. The graph illustrates the dynamic changes in the level of key reproductive hormones across a typical ovulatory menstrual cycle. Created in BioRender. Kuźmycz, O. and Wierzbińska, W. (2025) https://BioRender.com/es82lve.

While the number of immune cells in individual parts of the URT is relatively constant, the number of cells in the uterus is sensitive to changes in sex hormone levels caused by the ovarian cycle. Cyclical hormonal changes during the menstrual cycle may affect immune responses, which is crucial for promoting endometrial receptivity, necessary for embryo implantation (46). The normal ovulatory menstrual cycle depends on the proper functioning of a mature hypothalamic-pituitary-ovarian axis and the precise regulation of hormonal feedback mechanisms (**Figure 3**). The cycle is traditionally divided into two main phases: the follicular phase and the luteal phase, followed by menstrual bleeding. These phases are characterized by fluctuations in progesterone (P4), oestradiol (E2), follicle-stimulating hormone (FSH), and luteinizing hormone (LH) levels. Each cycle culminates in the development of mature follicles and the release of an oocyte, with menstruation occurring in the absence of fertilization. During the follicular phase, oestrogen levels gradually increase until a surge in LH secretion from the pituitary triggers ovulation. If fertilization does not occur, oestrogen levels rapidly decline within 24 hours. LH also prompts granulosa cells to shift from converting androgens to producing progesterone, leading to elevated progesterone levels throughout the luteal phase. In the absence of implantation, progesterone levels drop after 14 days, resulting in menstrual bleeding. This consistent pattern of a two-week estrogen-dominant environment, followed by a two-week progesterone-dominant phase during the female reproductive years, provides an opportunity to study the physiological role of these hormones in cellular homeostasis in an *in vitro* context (73, 74).

Experimental studies have shown that the immune cell composition in the endometrium undergoes dynamic changes during the menstrual cycle, particularly during menstruation. Immune cells, including T-helper cells (CD4+ T cells), cytotoxic T cells (CD8+ T cells), natural killer (NK) cells, and macrophages, fluctuate in response to hormonal changes. There is a marked infiltration of macrophages during menstruation, which play a key role in tissue remodeling and the clearance of apoptotic cells. Additionally, dendritic cells are involved in initiating immune responses and show dynamic changes in number and activation state throughout the menstrual cycle. These fluctuations of immune cells are tightly regulated by hormonal signals, primarily estrogen and progesterone, and contribute to maintaining immune homeostasis in the endometrium, which is essential for reproductive health. Both DCs and macrophages reach their peak number during the late secretory phase (75). Other cells playing an important role during menstruation are endometrial neutrophils. Through the elastase release resulting in the activation of extracellular metalloproteinase, neutrophils promote the destruction of endometrial tissue. This allows intimal remodeling and intimal vascular repair (62). The T cell level is subject to fluctuations during the menstrual cycle, representing 50% of the leukocyte population during the proliferative phase and decreases to <10% by the late secretory phase (76). NK cells, crucial for immunosurveillance, also exhibit increased activity during menstruation, contributing to regulating endometrial shedding and tissue repair. The T-regulatory cell levels (Tregs), which are involved in immune tolerance, are generally reduced during menstruation, reflecting the heightened inflammatory response necessary for endometrial breakdown. When it comes to endometrial mast cells, their abundance in the endometrium is relatively low, and the number of cells does not undergo significant changes during the menstrual cycle (63).

### 3.4 TLRs as specific microbiological identifiers

The survival of commensal microbiota within the endometrium is determined by the presence of Toll-like receptors (TLRs), which enable the differentiation of pathogenic microorganisms from the physiological microbiota, thus strengthening the endometrial defense barrier. These receptors can be found on the surface of columnar cells lining the upper FRT and on immune cells such as macrophages, dendritic cells, neutrophils or NK cells (77–79).

TLRs represent a group of trans-membrane receptors belonging to the family of pattern recognition receptors (PRRs). In their structure, one can differentiate an extracellular domain, a very short trans-membrane domain and a cytoplasmic domain. The extracellular domain is composed of leucine-rich repeats and is responsible for the binding of a well-defined ligand by a specific receptor. It plays a key role in recognizing pathogen-associated molecular patterns (PAMPs), such as bacterial or viral nucleic acids, LPS, lipoproteins, or peptidoglycan (80). The cytoplasmic domain, also known as the Toll/IL-1 receptor (TIR) domain due to its homologous nature to the cytoplasmic domain of the human IL-1 type 1 receptor, has cysteine units at its ends, which are responsible for initiating signaling cascades in the cell, ultimately leading to the activation of transcriptional nuclear factor κB (NF-κB), crucial for gene expression of a wide range of immune response-related molecules such as cytokines, chemokines, costimulatory molecules or adhesins (56, 81). Thus, the role of TLRs is not limited to a receptor function during the detection of molecular patterns of microorganisms but is also expressed through their key role in the activation of the inflammatory response through the secretion of pro-inflammatory cytokines. It is worth noting that cytokine secretion resulting from TLR receptor activation can also indirectly induce the differentiation of Th1-type lymphocytes, as well as activate monocytes and NK cells, which will involve the secretion of further inflammatory mediators such as interleukin-2 (IL-2), IL-12, IL-15, IL-18, tumor necrosis factor α (TNF-α), interferons α (IFN-α) and β (IFN**-**β**).** TLRs activated on the surface of B lymphocytes, in turn, lead to direct activation of these cells, resulting in their secretion of antibodies, which are precise tools to combat pathogenic microorganisms. TLR receptors thus play their role by combining specific and nonspecific immune responses (82–84). Among mammals, we identify 11 different TLR receptors, the presence of which within the endometrium for most of them has been confirmed by studies. Their expression in the uterine cavity is subject to changes depending on the woman’s physiological state or the phase of the menstrual cycle (85–87). In order to recognize a specific ligand pair or a specific microbial ligand TLRs can form homodimers, meaning they have two identical receptor chains or heterodimers consisting of 2 different receptor chains, which are formed by TLR-2 and TLR-1 or TLR-6. TLR1, TLR2, TLR4, TLR5, and TLR6 mainly recognize bacterial products present on the surface of microbial cells, whereas TLR3, TLR7, TLR8 and TLR9 tend to recognize nucleic acids (88, 89). Without a doubt, TLRs are a key element in the induction of inflammation through the detection and recognition of foreign molecular patterns, resulting in the activation of transcription factors responsible for the secretion of inflammatory mediators. However, their role is not limited to pathogen detection and control. TLR receptors also condition host immune tolerance to commensal microbiota inhabiting particular FRT niches. These microorganisms are recognized by TLRs, but the immune response is not directed against them. Thus, one can say that TLRs are also a kind of a specific identifier, enabling the survival of selected microorganisms within the endometrium (90, 91).

## 4 Dysbiosis in endometrial immunopathologies

### 4.1 Endometrial dysbiosis

In a situation where the microbial load is too high or rich in representatives of certain taxa, excessive tissue destruction and immune stimulation can occur, leading to an imbalance of homeostasis between bacteria, which is dysbiosis. It is not known whether dysbiosis is a cause or an effect of the disease. However, there is no doubt that disorders within the composition of the endometrial microbiome are associated with the occurrence of abnormalities in the physiology of the female reproductive system (18).

Disruption of the endometrial microbiota homeostasis may result in pathological conditions such as endometritis, defined as chronic inflammation caused by bacterial pathogens. Due to its asymptomatic course, it is an infrequently diagnosed condition that can be closely associated with recurrent implantation failure (RIF) or pregnancy loss. It is estimated that endometritis can affect up to 45% of infertile patients (92). Another disorder underlying dysbiosis in the reproductive system is endometriosis, characterized by the presence of endometrial tissue outside the uterine cavity, resulting in pelvic pain or infertility. It is believed that this disease affects up to 10% of women of reproductive age. The causes of the incidence of endometriosis are not yet fully understood, but it is thought that it may have a bacterial origin (93). This theory derives from a study showing increased incidence of the isolation of microorganisms from the genus *Actinomyces*, *Corynebacterium*, *Enterococcus*, *Fusobacterium*, *Gardnerella*, *Prevotella*, *Propionibacterium*, *Staphylococcus*, *Streptococcus* or *E. coli* from menstrual blood and endometrial samples of endometriosis patients, compared to a control group, in which mainly lactic acid bacteria were observed. Among the abnormalities underlying dysbiotic disorders in the uterine cavity, endometrial neoplasia is also receiving increasing attention. An enrichment of certain taxa has been observed among patients suffering from hyperplasia and cancer, suggesting an inflammatory role for certain bacteria in carcinogenesis (94).

### 4.2 Inflammation in the pathogenesis of EC

Endometrial cancer is currently the most commonly diagnosed malignancy affecting women in developed countries, and an increasingly problematic gynecological cancer. It is estimated that there were nearly 400,000 new cases worldwide in 2018, and according to the International Agency for Research on Cancer, the incidence rate of this cancer, by 2040, will increase by up to more than 50% worldwide (95).

Histologically, one can differentiate between 2 types of EC: estrogen-dependent (I type), which accounts for 10-20% of all cases, and non-estrogen-dependent (II type), which accounts for 80-90% (7). Among the risk factors for endometrial cancer, the most cited are high Body Mass Index (BMI), disorders related to hormonal imbalance, diabetes, or hypertension. Excessively high blood pressure can inhibit apoptosis, which can promote the growth of cancer cells. Studies also indicate a lower incidence rate of endometrial cancer among patients who have children. Hormonal changes during pregnancy usually result in increased production of progesterone, which is attributed to a protective effect against endometrial cells, which may account for the lower incidence of cancer among patients who have children. The most cited risk factor, however, is obesity, which is attributed to an inflammatory role that may contribute to the induction of neoplastic processes, as well as hyperinsulinemia resulting from excessive body weight, which is a common predecessor to diabetes. Persistently high blood insulin concentrations have mutagenic effects and can cause an increase in the body’s levels of bioavailable estrogens by decreasing the concentration of sex hormone-binding globulin (SHBG) (96). The effect of obesity is also very often caused by disturbances in the intestinal microbiota, which plays a significant role in the modulation of the reproductive endocrine system through interactions with insulin, androgens, or estrogens - hormones crucial for the carcinogenesis of the female reproductive tract, which can also affect the composition of the microorganisms inhabiting the uterine cavity.

In 1863, the prominent German pathologist Rudolf Virchow observed the presence of lymphocytes in tumor tissues, which allowed him to hypothesize that chronic inflammation links to the onset of cancer. More than 150 years later, we dispose of evidence that enables us to confirm that his hypothesis was correct (97). It is estimated that up to 13-20% of cancer cases diagnosed each year are associated with infections, the main effect of which is inflammation (98, 99). Usually, during an acute inflammatory response caused by the pathogens, complete elimination of microorganisms occurs, which contributes to the restoration of homeostasis. However, in some infections, elimination of the pathogen does not happen, and the secreted pro-inflammatory factors can promote a chronic inflammatory process that, if sustained over a long period, may pose a risk for cancer development. Microorganisms, including bacteria and viruses, have long been suspected of a potential role in cancer induction and development, but identifying the specific species that could be responsible for such processes and the underlying mechanisms has so far been a challenge. To date, the International Agency for Research on Cancer has classified only one bacterial species, *Helicobacter pylori*, as a human carcinogen (100). Infection with this microorganism can result in inflammation of the gastric mucosa, which in a chronic state can lead to peptic ulcer disease and, consequently, also cancer (101). A similar relationship has been demonstrated for *Fusobacterium* sp., particularly the species of *Fusobacterium mortiferum*, *Fusobacterium nucleatum* or *Fusobacterium necrophorum* and colon cancer. The *F. nucleatum* bacterium is responsible for activating the nuclear factor NF-κB, a key factor regulating the course of the inflammatory response, which in the dysbiosis stage can cause the development of chronic inflammation (102). Another study showed that in the case of this cancer, the genus *Porphyromonas*, too, can induce the process of carcinogenesis (103). Admittedly, numerous literature data highlight the key role of inflammation in the processes of the FRT, such as the menstrual cycle, ovulation, and implantation, emphasizing that the endometrium is a dynamic environment whose physiological function depends on cyclic processes in which inflammation is alternately induced to then become self-silenced. Unfortunately, when inflammation becomes a chronic condition, it can endanger a woman’s health (104).

The link between chronic inflammation has so far also been demonstrated for endometrial cancer. The role of inflammation in EC was first pointed out by Modugno et al. (105) who showed a link between the inflammatory environment in endometrial tissue, the production of inflammatory mediators, and the development and progression of endometrial cancer. In a case-control study performed by Doddus et al. (106) in 2011, it was shown that one of the key inflammatory mediators, TNF, was responsible for proliferation and involved in angiogenesis of the tumor cells studied. In another study, Dossus et al. (107) examined the relationship between the presence of various biomarkers such as hormones, growth factors and cytokines in the pre-diagnostic blood of patients and the occurrence of endometrial cancer. The study demonstrated an association between the presence of TNF-α, TNF receptors 1 and 2, IL-6, and C-reactive protein (CRP) and an increased risk for endometrial cancer in postmenopausal women. Another study by Dossus et al. (108) showed a strong association between elevated cytokine levels and increased risk of endometrial cancer, thus indicating that endometrial cancer may have an inflammatory basis. A similar relationship was demonstrated in a study by Trabert et al. (109), in which the levels of 64 inflammatory biomarkers in the blood were evaluated and showed that EC risk was highest among women with the highest levels of inflammatory markers including adipokines, inflammatory cytokines, angiogenic factors, acute phase proteins and vascular endothelial growth factor-A (VEGF-A). The study by Liu et al. (110) collected and sequenced endometrial biopsy specimens from 130 infertile women with confirmed chronic endometritis and showed that malignancy was not only associated with chronic inflammation but also with an increased abundance of bacterial taxa such as *Dialister*, *Bifidobacterium*, *Prevotella*, *Gardnerella* and *Anaerococcus*, shedding new light on the role of microorganisms in the induction of inflammation during EC.

### 4.3 TLRs and mediators of inflammation in the tumor progression

**Figure 4:**
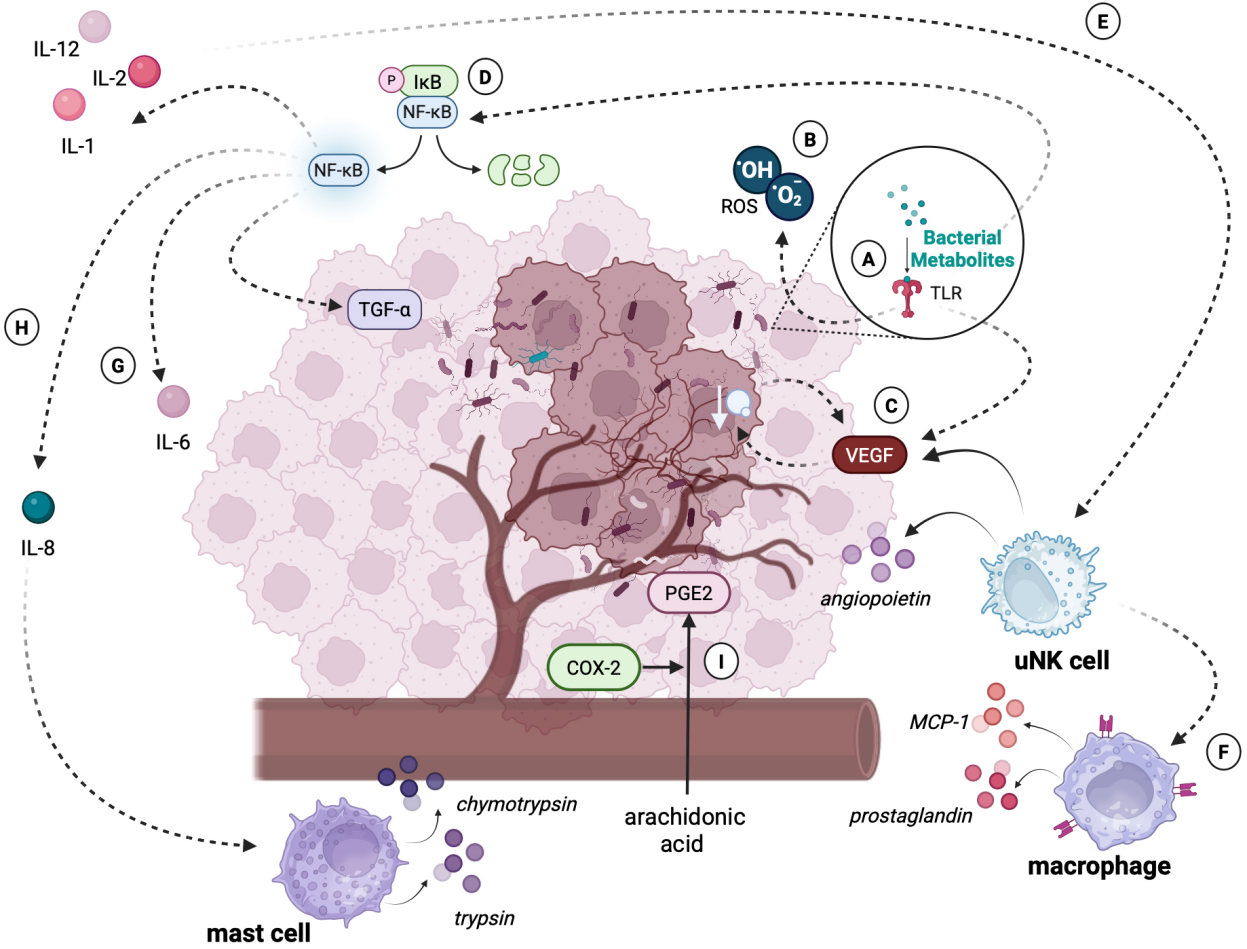
An excessive immune response of the tumor microenvironment caused by bacterial dysbiosis. The figure illustrates the molecular and cellular mechanisms through which bacterial dysbiosis contributes to the progression of endometrial cancer: **(A)** Detection of bacterial metabolites or PAMPs by TLRs triggers the production of ROS and activates signaling pathways such as NF-κB. These pathways lead to the secretion of pro-inflammatory cytokines (e.g., IL-6, IL-8) and angiogenic mediators. **(B)** ROS play a dual role as signaling molecules regulating processes of transcription, differentiation, and apoptosis. However, excessive ROS levels cause DNA damage, genomic instability, and mutations, creating a microenvironment conducive to tumor transformation. **(C)** Production of VEGF promotes angiogenesis, allowing tumor cells to access nutrients and oxygen. The newly formed vessels are structurally abnormal, leading to tissue hypoxia, which further stimulates VEGF production and exacerbates tumor progression. **(D)** NF-κB activation induces a wide range of pro-inflammatory cytokines (e.g., IL-6, IL-8) and mediators (e.g., TGF-β), which enhance metastasis, tumor cell proliferation, and angiogenesis. **(E)** Activated uNK cells, stimulated by IL-2 and IL-12, contribute to angiogenesis by increasing VEGF synthesis and releasing angiopoietins. **(F)** Macrophages, driven by chemokines such as MCP-1 and prostaglandins (e.g., PGE2), intensify inflammatory processes, further destabilizing the tumor microenvironment. **(G)** IL-6, which secretion is also a result of NF-κB metabolic pathway activation, enhances the proliferation, migration and invasion of endometrial cancer cells. **(H)** IL-8 overexpression facilitates leukocyte recruitment, including mast cells. These cells release proteolytic enzymes (e.g., trypsin, chymotrypsin), contributing to tissue remodeling and cancer invasion. **(I)** COX-2, upregulated during chronic inflammation, catalyzes the conversion of arachidonic acid into prostaglandins (e.g., PGE2). These compounds enhance the inflammatory response, inhibit apoptosis, and promote angiogenesis, further supporting tumor development. Created in BioRender. Wierzbińska, W. (2025) https://BioRender.com/9rwu2yc.

Certain bacteria in the endometrial environment may play a key role in the initial stages of inflammation by inducing an excessive inflammatory response (**Figure 4**) (111). Molecular patterns of pathogenic bacteria stimulate the activation of metabolic pathways by TLR receptors, resulting in the production of pro-inflammatory cytokines. Under physiological conditions, activation of the inflammatory response by receptors is a favorable situation, as it allows our immune system to fight off the intruder. However, in chronic dysbiosis, TLRs may co-create a tumor-promoting environment by initiating and enhancing the inflammatory response or promoting cellular angiogenesis (112). Experimental studies have shown that Toll-like receptors, the key components of the innate immune system, play a significant role in the early stages of tumorigenesis in EC. TLRs, particularly TLR-4, are involved in the recognition of pathogen-associated molecular patterns (PAMPs) and damage-associated molecular patterns (DAMPs), which initiate the inflammatory response. This activation leads to the upregulation of pro-inflammatory cytokines, such as TNF-α and IL-6, contributing to development of the microenvironment that promotes tumor cell adhesion and invasion (113). Specifically, TLR-4 activation has been implicated in enhancing the adhesion of endometrial cancer cells to the extracellular matrix and epithelial cells, facilitating their early attachment and colonization (114). Additionally, TLR signaling has been shown to influence the recruitment of immune cells to the site of infection or tumor development, further promoting the inflammatory milieu that supports cancer progression (115). These findings suggest that TLRs are not only involved in immune responses but may also act as key mediators in the initiation and progression of endometrial cancer.

Activated TLRs may also play a key role in angiogenesis by stimulating the secretion of VEGF, which promotes tumor vascularization. The presence of blood vessels enables significant tumor growth because of the nutrients and oxygen supplied. However, the vessels formed under the influence of VEGF are characterized by both functional and structural differences, compared to those found in healthy tissues. These differences result in increased hypoxia of the tumor-affected tissue. Hypoxia, in turn, stimulates further VEGF synthesis and tumor growth (116). The relationship between increased synthesis of VEGFs along with selected TLRs and the degree of tumor progression was confirmed in a study conducted on a sample of 123 neoplastic endometrial samples, which managed to show a significant correlation between the expression of selected TLRs along with VEGRs and the degree of clinical progression and tumor pathology (117).

Recognition of bacterial molecular patterns by TLR receptors may also result in overactivation of NF-κB. NF-κB is a protein complex involved in the synthesis of many pro-inflammatory cytokines, playing a key role in regulating the inflammatory response. However, the ability of the NF-κB factor to inhibit apoptosis, induce proliferation, and enhance angiogenesis suggests its important role in tumor progression (118). Indeed, a study using immunohistochemical methods conducted on a group of 59 patients diagnosed with endometrial cancer, benign endometrial pathology, and with endometrial hyperplasia showed that the group with EC had significantly higher levels of NF-κB factor expression, compared to the other groups (119). Another study on a group of patients with endometrial cancer showed that the group of cancer patients had significantly higher serum levels of NF-κB, compared to the group of healthy patients (120).

Cytokines are a diverse group of soluble proteins released mainly by cells of the immune system in response to infection, inflammation, or tissue damage. To date, the secretion of the cytokines IL-6, IL-17, as well as transcription of genes coding inflammatory factors such as IL-8 (121) has been observed in tumor-lesioned endometrial tissues in which the presence of microorganisms has been confirmed. IL-6 is one of the main factors involved in the inflammatory response against pathogens. It plays an important role in the immune response, induction of inflammation, hematopoiesis, and progression of many cancers, including gynecological cancers. A study showed that the presence of IL-8 and IL-6 in the ovarian cancer setting was significantly correlated with a poorer prognosis of patients (122). This relationship was also confirmed in endometrial cancer. A study by Che et al. (123) showed that the presence of IL-6 in the tumor cell environment correlates with the invasion and migration abilities of endometrial cancer cell lines, suggesting a role for cytokines in tumor progression. Another study showed that IL-6 indirectly stimulates the proliferation, migration, and invasion of endometrial cancer cells and downregulates the expression of protooncogenes in cancer cells (124). IL-8 also plays an important role in tumor angiogenesis in the course of breast, ovarian, cervical, and also endometrial cancers. Overexpression of IL-8 causes infiltration of large numbers of leukocytes to the tumor by chemotaxis. Attracted immune cells release growth factors in the tumor microenvironment, thus promoting tumor growth and proliferation (125). A study by Ciortea et al. (126) found significantly elevated levels of IL-8 in a group of 44 patients with endometrial cancer compared to a group without gynecologic pathology.

Activation of TLR receptors, including TLR-2, also contributes to the induction of oxygen free radicals (ROS) (127). ROS are a group of byproducts of normal cellular metabolism such as superoxide anion (O^2-^), hydrogen peroxide (H_2_O_2_) and hydroxyl radical (OH^-^). Maintained in a physiological state, ROS act as signaling molecules that regulate the course of numerous reactions, including growth factor signaling, proliferation or adaptation to hypoxia (128). However, excessive levels of ROS in the cellular environment can have pathological effects. Indeed, ROS play a key role in post-transcriptional gene regulation, modulating cellular development, differentiation, proliferation, and apoptosis, being some of the key inflammatory mediators in cancer progression (129). Indeed, a study of 305 EC patients showed that their ROS levels were significantly higher than those of healthy controls (130). In addition, the results of a recent Lopez-Mejia study suggest that oxidative stress is increased in uterine serous carcinoma (USC), a more aggressive form of EC, and promotes metastasis (131). Chronic inflammatory response within the endometrium can also lead to overexpression of a wide range of inflammatory factors and mediators, including the enzyme cyclooxygenase-2 (COX-2). Under physiological conditions, this enzyme is a key component in the prostaglandins (PGs) synthesis, including prostaglandin E2 (PGE2), formed by the COX-2-induced metabolism of arachidonic acid. Synthesis of PGE2 leads to increased metabolic and inflammatory processes, as well as inhibition of apoptosis, which may result in the initiation of angiogenesis, leading to tumor formation (132). Also, the overgrowth of certain strains within the uterine cavity, including *G. vaginalis*, the presence of which within the tumor-transformed endometrium is confirmed by several studies (121, 133–140), can lead to overexpression of cyclooxygenase-2. In addition to its ability to initiate COX-2 synthesis, the bacterium is also responsible for vaginolysin (VLY) synthesis, a bacterial toxin that can serve as an inflammation-inducing agent and exhibit promiscuous activity against epithelial cells (141).

### 4.4 Genome destabilization in EC immunopathogenesis

Dysbiotic disorders within the endometrial microbiota and persistent chronic inflammation can also lead to the destabilization of the host genome. Chronic inflammation resulting from a disruption of microbial homeostasis within the endometrium provides an environment that promotes damage to the host’s genetic material. Metabolic changes resulting from mutations or damage to host DNA can result in cell cycle disruption, inhibition of apoptosis or stimulation of proliferation of malignant cells (142, 143). Among other things, the changes can be associated with overexpression of COX1 and COX2 enzymes, nitric oxide, and ROS. High concentrations of these factors in the tumor microenvironment promote damage to the genetic material of host cells, which can result in the formation of mutations (144). Microorganisms can also cause damage to host genetic material by secreting products of secondary metabolism, including toxins, or altering the pH of the environment (2). According to the model proposed by Knudson, carcinogenesis requires at least two allelic mutations in a single gene. These mutations include genes involved in cell cycle control or genomic DNA quality. Such single mutations could, of course, lead to the development of cancer, but more mutations are usually required. Chronic inflammation provides a highly mutagenic environment that promotes changes at the epigenetic level. The resulting mutations can lead to abnormalities in cellular metabolism, such as proliferation or apoptosis, processes crucial to the course of carcinogenesis (144, 145).

Differences in the metabolism of cancer cells compared to unaltered tissue were demonstrated in 2017 by Altadill and colleagues (146), who compared the metabolomics of EC tissue samples from 39 patients and tissues from a sample of 17 healthy women. They observed abnormalities in lipids, kynurein, endocannabinoids and the RNA editing pathway in cancer patients. Further studies, in turn, showed that adenosine deaminase acting on RNA2 (ADAR2) is overexpressed in cancer cells, possibly contributing to carcinogenesis. Unfortunately, this study does not indicate whether these changes may be the effect of the presence of pathogenic microorganisms within the tumor-lesioned tissue. Another study by Chen et al. (137) provided ample evidence for the role of microorganisms in modulating the metabolic pathways of host cells. In the first part of the study, the team showed elevated levels of representatives of the genera *Firmicutes*, *Proteobacteria*, *Tenericutes*, *Actinobacteria*, and *Bacteroidetes* among EC patients, and, even more significantly, the microorganisms participated in the metabolic conversion of N-acetyl-β-glucosaminyl to 6-sulpho-sialyl Lewis x epitope and promoted the 6-sulpho-biosynthesis of sialyl Lewis x epitope, a molecule responsible for promoting metastasis by adhering tumor cells to endothelial cells. The study was also the first to confirm the presence of these molecules in the endometrium. In addition, the study proved that the microorganisms whose increased presence was noted in tumor-lesioned EC tissue had an inhibitory effect on N-Glycan synthesis and were associated with activation of the Apelin signaling pathway, whose association with increased risk of EC was confirmed in a study by Yang et al. (147). The results presented not only indicate the role of microbes in modulating the metabolism of host endometrial cells but also provide interesting data on the potential role of dysbiotic disorders in metastasis. The evidence presented here suggests that the potential role of microorganisms in the induction of tumorigenic processes should not be downplayed.

## 5 Dysbiosis in EC

### 5.1 Endometrial dysbiosis in EC

**Figure 5:**
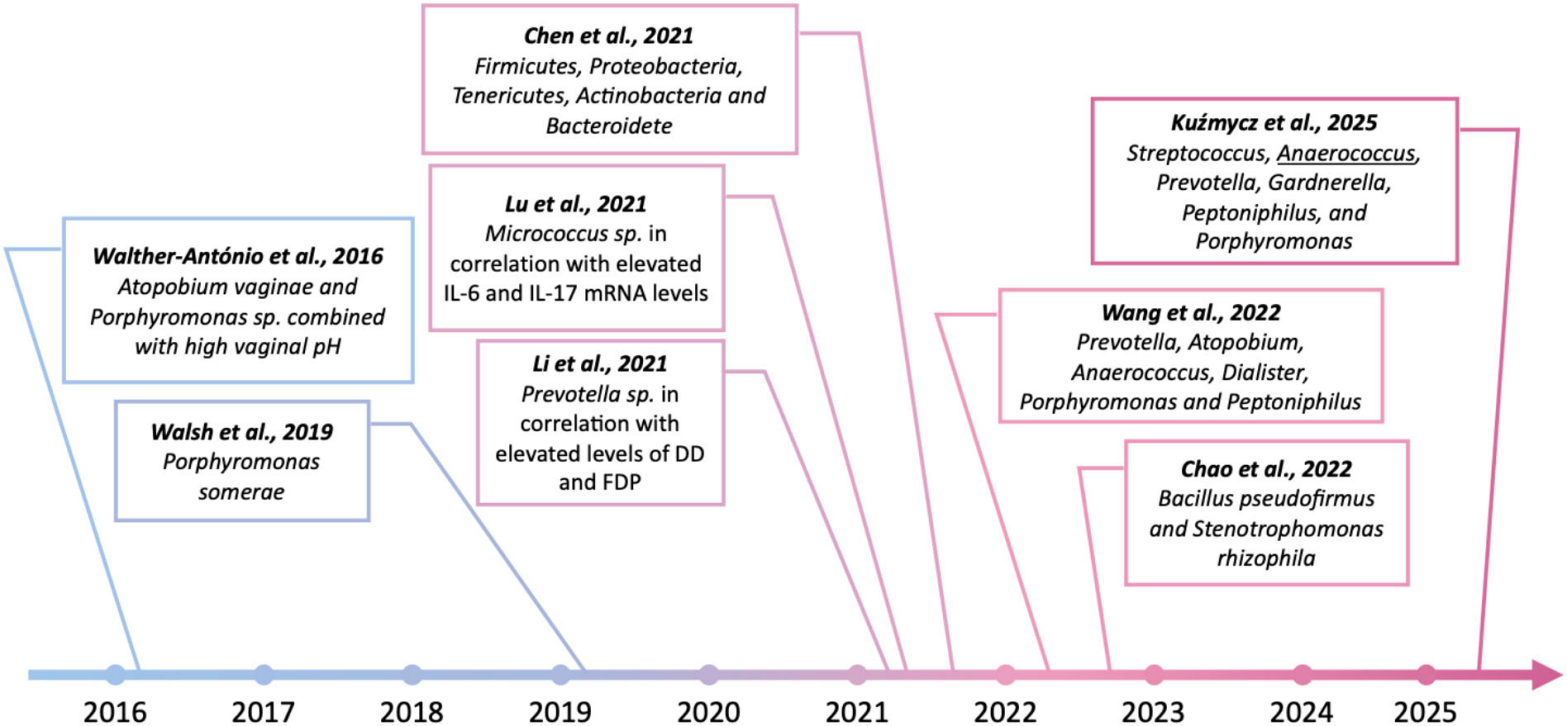
Findings indicating the potential role of pathogenic bacteria in the development of endometrial cancer over the past 10 years. Created in BioRender. Wierzbińska, W. (2025) https://BioRender.com/fqwiu4m.

The correlation between endometrial dysbiosis and endometrial cancer is indicated by the results of studies from the last decade (**Figure 5**). The link between the occurrence of EC and the presence of specific bacterial strains within the altered tissues was first demonstrated by Walther-António (133). The experiment was conducted on a group of 17 women who had undergone a hysterectomy for an established cancer. The results of next-generation sequencing of the 16S rDNA V3-V5 region revealed that the presence of *Atopobium vaginae* and *Porphyromonas* sp. bacteria in combination with high vaginal pH was statistically associated with endometrial cancer.

In another study of 148 women undergoing hysterectomy for benign lesions, endometrial hyperplasia or endometrial cancer, Walsh et al. (134) also identified *Porphyromonas somerae* as the most predictive microbiological marker of endometrial cancer. Moreover, Caselli et al. (135) showed that *P. somerae* and *A. vaginae* induced the release of pro-inflammatory cytokines by human endometrial cells after 24 hours of co-culture. As indicated by the results of the above studies, enrichment in certain taxa was observed among both hyperplasia and cancer patients, suggesting an inflammatory role of specific microorganisms in carcinogenesis. Nowadays, a growing number of studies point to the presence of particular strains as potential biomarkers of endometrial cancer. In the stage of dysbiosis, microorganisms, especially certain pathogenic taxa, can stimulate immunopathological processes and promote destabilization of the host genome. In a recent study, Li et al. (136) confirmed that increasing *Prevotella* abundance in endometrial tissue, specifically when correlated with elevated serum levels of D-dimers (DD) and fibrin degradation products (FDP), may be an important factor associated with carcinogenesis. A study by Lu et al. (121) found a correlation between increased *Micrococcus* abundance and IL-6 and IL-17 micro-RNA (miRNA) levels in endometrial cancer patients, suggesting a pro-inflammatory role for these microorganisms in carcinogenesis. Another study indicated significant enrichment of representatives of the genera *Firmicutes*, *Proteobacteria*, *Tenericutes*, *Actinobacteria*, and *Bacteroidetes* in the tumor-lesioned endometrium of EC patients (137). In contrast, in a study conducted on a group of 28 postmenopausal women undergoing hysterectomy, Wang et al. (138) showed that although *Lactobacillus* and *Gardnerella* were the predominant taxa in both tumor tissue samples and adjacent non-tumor tissue, only the group of sections taken from cancer patients was enriched in *Prevotella*, *Atopobium*, *Anaerococcus*, *Dialister*, *Porphyromonas* and *Peptoniphilus*. An interesting result was revealed by Chao et al. (139), whose study showed an increased relative abundance of plastic-degrading bacteria, *Bacillus pseudofirmus* and *Stenotrophomonas rhizophila*, in endometrial washings from patients with EC or endometrial hyperplasia. In a recent study, Kuźmycz et al. (140) not only found that the group of EC patients exhibited higher microbial diversity compared to the control group but also indicated higher abundance of pathogenic taxa such as *Streptococcus, Anaerococcus, Prevotella, Gardnerella, Peptoniphilus*, and *Porphyromonas* in malignant samples. In this study, the authors emphasized the role of *Anaerococcus*, which has emerged as a taxon that differentiates microbiomes isolated from women with EC and those with benign lesions, possibly representing a pathogenic cofactor of EC and at the same time a potential biomarker of carcinogenesis process. The above results suggest a potential role of selected pathogens in the induction of inflammatory-mediated carcinogenesis processes in EC. However, due to the heterogeneity of the study procedures and the results obtained, we cannot yet conclusively determine whether and how microorganisms induce pathological processes within the uterine cavity (**Table 2**). However, there is no doubt that dysbiotic disorders within the endometrium should not be underestimated.

**Table 2:** The comparison of the methodologies and key findings in studies of EC microbiota.

Study	Sampling method	Sequencing techniques	Target region	Key findings
Walther-António et al., 2016 (133)	endometrial biopsy samples from women with endometrial cancer (EC) and controls	16S rRNA amplicon sequencing	V3 - V5	presence of *Atopobium vaginae* and *Porphyromonas* sp. bacteria in combination with high vaginal pH was statistically associated with endometrial cancer.
Walsh et al., 2019 (134)	endometrial tissue samples from postmenopausal women with and without EC	16S rRNA amplicon sequencing	V3 - V5	*P. somerae* emerged as the most significantly enriched species in patients with EC. In addition, EC patients were significantly more likely to have a high vaginal pH.
Li et al., 2021 (136)	endometrial tissue and paired blood samples from EC patients and healthy controls	Shotgun metagenomic sequencing	Not applicable	Increased *Prevotella* abundance in endometrial tissue, specifically when correlated with elevated serum levels of D-dimers (DD) and fibrin degradation products (FDP), may be an important factor associated with carcinogenesis.
Lu et al., 2021 (121)	endometrial tissue from EC patients and healthy controls	16S rRNA amplicon sequencing	V3-V4	Correlation between increased *Micrococcus* abundance and IL-6 and IL-17 micro-RNA (mRNA) levels in endometrial cancer patients.
Chen et al., 2021 (137)	endometrial biopsies from EC patients and healthy controls	metatranscriptome (meta-RNA sequencing analysis)	Not applicable	Top five phyla that were significantly enriched in the endometrium of patients with endometrial cancer were *Firmicutes, Proteobacteria, Tenericutes, Actinobacteria*, and *Bacteroidetes.*
Wang et al. (138)	endometrial and pericancer tissue samples from postmenopausal women with EC	16S rRNA amplicon sequencing	V3-V4	Although *Lactobacillus* and *Gardnerella* were the predominant taxa in both tumor tissue samples and adjacent non-tumor tissue, only the group of sections taken from cancer patients was enriched in *Prevotella*, *Atopobium*, *Anaerococcus*, *Dialister*, *Porphyromonas* and *Peptoniphilus.*
Chao et al. (139)	endometrial lavage samples from women with endometrial cancer and endometrial hyperplasia	16S rRNA amplicon sequencing	V3-V4	Increased relative abundance of plastic-degrading bacteria, *Bacillus pseudofirmus* and *Stenotrophomonas rhizophila*, in endometrial washings from patients with EC or endometrial hyperplasia.
Kuźmycz et al. (140)	endocervical canal swabs from patients qualified for surgical treatment due to diagnosed EC or endometrial myoma (EM)	16S rRNA amplicon sequencing	V3-V4	Pathogenic taxa such as *Streptococcus, Anaerococcus, Prevotella, Gardnerella, Peptoniphilus*, and *Porphyromonas* were enriched in EC samples. Genus *Anaerococcus* was found to be a differentiating taxon between microbiomes isolated from women with EC and those with benign lesions.

### 5.2 Gut microbiota dysbiosis in EC

Over the past 20 years, our knowledge of the human microbiome and its role in physiology has increased significantly. Research into the composition and function of microorganisms residing in various niches of the human body proved that the microbiota plays an extremely important role in our physiology. Particular importance in the proper functioning, but also in the pathogenesis of many diseases, has been attributed in recent years to gut microbiota (148, 149).

Balanced gut microbiota is composed of a diverse group of microorganisms belonging to 4 main phyla: *Bacteroidetes, Firmicutes, Actinobacteria* and *Proteobacteria*, from which more than 90% of species consist of *Bacteroidetes* and *Firmicutes* phyla (150). A balance between commensal microbes inhabiting the gut is essential for their proper function in maintaining the epithelial barrier, improving digestion and regulating adaptive and innate immune responses (151, 152). The epithelial barrier is described as a monolayer of cells joined through protein complexes which separates the commensal bacteria in the gut from the underlying tissues. Additional protection against excessive microbial invasion is provided by mucus covering the epithelial layer. Its production and degradation are susceptible to changes in the composition of gut microbiota (153). Both inflammation and changes in the composition of the local microbiota can contribute to disturbances in mucus secretion and composition, leading to increased susceptibility to infection. Interaction between gut microbiota and intestinal epithelium is enabled by PRRs. Their presence leads to continuous immune signaling, the regulation of which is essential for maintaining intestinal homeostasis (154).

The intestinal microbiota is also responsible for the production of short-chain fatty acids (SCFAs) - metabolites produced in the process of fermentation of dietary fibers, proteins, and other substrates. By lowering the intestinal pH, SCFAs promote the growth of beneficial bacteria, such as Lactobacilli and *Bifidobacteria*, while inhibiting the colonization of pathogenic microorganisms like *Clostridium* and *Escherichia coli* (155). SCFAs also support the integrity of the intestinal barrier by stimulating the regeneration of epithelial cells and the production of mucus and antimicrobial peptides, which help prevent the translocation of toxins and pathogens into the bloodstream. Furthermore, SCFAs modulate the immune response by promoting the maturation and expansion of Tregs in the colon, which suppress local inflammatory reactions to the microbiota. They also play a role in the proliferation of innate lymphoid cells (ILC3), which release IL-22, a cytokine crucial for the production of antimicrobial molecules by epithelial cells (156). Through these mechanisms, SCFAs contribute to protecting against inflammation, metabolic disorders, and infections, thereby playing a central role in gut health and immune regulation (157, 158). However, the impact of the gut microbiome has a significant impact not only on the gastrointestinal system but also on other physiological systems in the body (159). It has been shown to influence the central nervous system (CNS), where gut-derived metabolites and immune signaling pathways modulate brain function and behavior, a phenomenon known as the gut-brain axis (160, 161). Microbiota also plays a crucial role in regulating the immune system, affecting innate and adaptive immune responses (161). Moreover, gut microbes influence metabolic processes, and the growing evidence suggests their prominent role in affecting cardiovascular health by modulating blood pressure and cholesterol levels (162). Thus, the microbiota represents a complex and integral component that interacts with multiple organ systems, shaping overall health. Unfortunately, the systemic role of the microbiota in shaping the physiological homeostasis of so many systems also means that any disturbance within the gut microbial balance can cause disorders within systems seemingly unrelated to the digestive system (163). Studies indicate that imbalances in the gut microbiota can directly or indirectly lead to numerous pathologies, including depression, hypertension, atherosclerosis, rheumatoid arthritis and even cancer (164, 165). By affecting lipid metabolism and insulin sensitivity, it can also contribute to the development of conditions like diabetes and obesity – one of the main risk factors in the immunopathogenesis of EC (166). Obesity has been identified as a significant risk factor for the development of endometrial cancer through several biological mechanisms. These mechanisms include chronic hyperinsulinemia, elevated insulin-like growth factor (IGF) levels, and increased estrogen production (96). Hyperinsulinemia reduces the levels of IGF-binding proteins (IGFB1 and IGFB2), leading to higher free IGF-1 bioavailability, which in turn promotes mitogenesis and inhibits apoptosis in endometrial cells. Additionally, increased estradiol levels not only stimulate endometrial cell proliferation and prevent cell death but can also enhance local IGF-1 production in the endometrial tissue (167). Moreover, hyperinsulinemia can exacerbate tumorigenesis in estrogen-sensitive tissues by decreasing the concentration of sex hormone-binding globulin, which increases the bioavailability of estrogens (168). Estrogen therapy, which is commonly used to manage menopausal symptoms, has been associated with an increased risk of endometrial cancer due to its stimulatory effects on the endometrial lining (169). Recent research also suggests that the gut microbiota may play an indirect role in endometrial cancer development, possibly by influencing metabolic and immune pathways that interact with hormonal regulation and tumorigenesis (170).

### 5.3 Estrobolome in EC

The gut microbiome, particularly a subset of microbial genes known as the estrobolome, plays a pivotal role in regulating estrogen metabolism. The estrobolome consists of microbial genes, products of which are capable of metabolizing estrogens, primarily through the enzymatic action of β-glucuronidase. Estrogens, predominantly produced by the ovaries and adipose tissue, circulate in the body, mostly in a conjugated form. In this state, they are bound to glucuronic acid or sulfate, which renders them inactive and unable to bind to estrogen receptors. However, the action of microbial enzymes, specifically β-glucuronidase, deconjugates these estrogens, releasing the free, bioactive form capable of binding to estrogen receptors located throughout various tissues, including the endometrium (157). This enzymatic activity is crucial because only the unbound, free estrogens are biologically active and capable of exerting their physiological effects on cells.

In the gut, the microbiome influences the bioavailability of estrogen by modulating the process of conjugation and deconjugation. The estrobolome is, therefore, a key player in determining the balance of active estrogen in the body, with direct implications for hormonal regulation (161, 171). When the gut microbiome is in balance, the production of β-glucuronidase is tightly controlled, leading to a healthy level of active estrogen. However, dysbiosis can lead to an overproduction of β-glucuronidase, which increases the conversion of conjugated estrogens into their free, active forms. This shift can result in elevated levels of estrogen in circulation, which may stimulate excessive cell proliferation and inhibit apoptosis processes (144). In the context of endometrial cancer, this microbial-driven estrogenic activity is significant.

Increased estrogen levels, whether due to endogenous sources like adipose tissue in obesity or hormonal therapies, can lead to endometrial hyperplasia, a precursor to endometrial carcinoma (172). The role of the estrobolome in controlling the availability of bioactive estrogen underscores the intricate connection between the gut microbiome and the risk of developing hormone-related cancers. Disruption in the estrobolome’s activity can create an environment in which estrogen levels are abnormally high, promoting the initiation and progression of endometrial cancer (161, 162). Furthermore, the estrobolome may also influence the broader gut-vaginal microbiome axis, with changes in microbial composition in the gut potentially altering the vaginal microbiota and influencing the local estrogen metabolism in the reproductive tract (173). This interconnected microbial environment can synergistically affect the production of pro-inflammatory cytokines such as IL-6 and TNF-α, which in turn upregulate enzymes involved in estrogen synthesis, creating a feedback loop that sustains elevated estrogen levels. Such changes in estrogen homeostasis are a key factor in the development and progression of endometrial cancer (114). Indeed, study by Beratis et al. indicated that the increase in β-glucuronidase titer, as well as its enzymatic activity, corresponded to the process of clinical deterioration in patients with gynecologic cancer or pelvic inflammatory disease compared to the healthy control group (174). In another study, using Mendel randomization analysis, specific taxa of gut bacteria were shown to be significantly associated with the risk of developing EC. Strains belonging to the *Erysipelotrichaceae* and *Bifidobacteriaceae* families, among others, known to produce enzymes such as β-glucuronidase, correlated with increased EC risk, which may be related to their involvement in estrogen reactivation. These findings suggest that the gut microbiota - through enzymatic activity affecting hormonal metabolism - may play an important role in the pathogenesis of endometrial cancer (175). Another study showed that endometrial dysbiosis, characterized by reduced *Lactobacillus* abundance and increased β-glucuronidase activity, is associated with enhanced estrogen receptor ERβ expression and increased levels of pro-inflammatory cytokines. Since β-glucuronidase is involved in estrogen reactivation, its excessive activity may promote inflammation and endocrine signaling disruption in the endometrium. These findings suggest that disruption of the microbiota and estrobolome function may interact in the pathogenesis of estrogen-dependent diseases, including potentially EC (176). Therefore, understanding the role of the estrobolome and its influence on estrogen metabolism provides important insights into the pathogenesis of cancer and highlights potential therapeutic avenues that target the microbiome to modulate estrogen levels and reduce cancer risk.

## 6 Conclusions

Considering current research, the growing body of evidence suggests that microbial dysbiosis, both in the endometrial environment and in the gut, plays a critical role in the immunopathology of endometrial cancer. An excessive load of pathogenic microorganisms can trigger a cascade of inflammatory responses due to the activation of Toll-like receptors and the subsequent production of proinflammatory cytokines like IL-6 and IL-8. This persistent inflammation contributes to an environment conducive to tumor progression, characterized by angiogenesis, oxidative stress, and DNA damage, which ultimately supports carcinogenesis. The upregulation of TLRs, NF-κB, and cyclooxygenase enzymes further highlights the importance of chronic inflammation in EC pathogenesis. Moreover, the gut microbiome’s impact on systemic inflammation and estrogen metabolism, particularly through the estrobolome, is a key link to EC development. Dysbiosis in the gut can disrupt the balance of microbial enzymes, such as β-glucuronidase, leading to the increased bioavailability of active estrogens. This hormonal imbalance, in combination with obesity-related factors, can drive endometrial hyperplasia and promote the initiation of cancer. The interaction between gut-derived metabolites, immune signaling, and estrogen regulation underscores the interconnectedness of the microbiota in shaping both immune responses and hormonal homeostasis, key factors in EC risk. All these arguments strongly support the importance of dysbiosis in the immunopathogenesis of EC. However, the available literature data on the role of EC microbiota in tumor initiation and progression features a wide variety of research protocols used and consequently, results obtained. Standardization of the research protocols would allow studies to be conducted with greater replicability and accuracy, which is crucial for understanding the role of microorganisms in the pathogenesis and progression of EC. More research is needed not only to identify the molecular basis of microbial activity in this niche but also to understand the mechanisms that regulate it. Restoring microbial balance in both the reproductive tract and the gut may provide a promising approach to modulate the inflammatory and hormonal environments that drive EC. Targeting microbial dysbiosis through diet, probiotics, or other microbiome-directed therapies could offer new avenues for the prevention and treatment of this hormonally driven malignancy. Further research into the specific microbial species involved, their mechanisms of action, and their influence on host metabolism and immune responses will be essential to developing such interventions. New possibilities and strategies for EC prevention and therapy support, as well as new diagnostic techniques, may also result from this knowledge.

## Glossary

TME: tumor microenvironment
URT: upper reproductive tract
FRT: female reproductive tract
HMP: Human Microbiome Project
NGS: next generation sequencing
DC: dendritic cells
NK: natural killer cells
uNK: uterine natural killer cells
pNK: peripheral blood NK cells
IFN-γ: interferon γ
TNF-α: tumor necrosis factor α
GM-CSF: granulocyte-macrophage colony-stimulating factor
VEGF: vascular endothelial growth factor
PIGF: placental growth factor
APC: antigen presenting cell
uDC: uterine dendritic cell
MHC: major histocompatibility complex
IL: interleukin
PG: prostaglandin
MCP-1: monocyte chemoattractant protein-1
LPS: lipopolysaccharide
HBD-2: human beta-defensin-2
FasR: FasL-CD95 ligand
TLRs: Toll-like receptors
PRRs: pattern recognition receptors
PAMPs: pathogen-associated molecular patterns
TIR: Toll/IL-1 receptor
NF-κB: nuclear factor kappa B
LD: *Lactobacillus*-dominant
NLD: non-*Lactobacillus*-dominant
RIF: recurrent implantation failure
DD: D-dimers
DAMPs: damage-associated molecular patterns
FDP: fibrin degradation products
miRNA: micro-RNA
BMI: Body Mass Index
SHBG: sex hormone binding globulin
CRP: C-reactive protein
VEGF-A: vascular endothelial growth factor-A
ROS: oxygen free radicals
O₂⁻: superoxide anion
H₂O₂: hydrogen peroxide
OH^-^: hydroxyl radical
USC: uterine serous carcinoma
COX-2: cyclooxygenase-2
PG: prostaglandin
PGE₂: prostaglandin E₂
VLY: vaginolysin
ADAR2: adenosine deaminase acting on RNA2
IGF: insulin-like growth factor
ILC3: innate lymphoid cells
CNS: central nervous system
